# Breastfeeding women in need of information about antiemetics for nausea and vomiting during pregnancy: a review of inquiries to a medicines information service

**DOI:** 10.3389/fphar.2023.1238875

**Published:** 2023-11-29

**Authors:** Kristine Heitmann, Tina Bakkebø, Gro C. Havnen, Jan Schjøtt

**Affiliations:** ^1^ Regional Medicines Information and Pharmacovigilance Centre (RELIS), Department of Clinical Biochemistry and Pharmacology, Haukeland University Hospital, Bergen, Norway; ^2^ Department of Clinical Science, Faculty of Medicine, University of Bergen, Bergen, Norway; ^3^ Regional Medicines Information and Pharmacovigilance Centre (RELIS), Oslo University Hospital, Oslo, Norway

**Keywords:** hyperemesis gravidarum, nausea and vomiting during pregnancy, breastfeeding, medicine safety, medicines information

## Abstract

**Introduction:** The medicines information service, SafeMotherMedicine, regularly receives inquiries from breastfeeding women asking about antiemetics for nausea and vomiting during pregnancy (NVP) or hyperemesis gravidarum (HG). However, treatment guidelines for NVP or HG do not address the use of antiemetics in women who are breastfeeding while becoming pregnant again. Our objective was to characterize inquiries to describe the need for lactation risk information among women with NVP or HG and also to raise awareness of this topic.

**Method:** We conducted a review of inquiries to the Norwegian web-based medicines information service, SafeMotherMedicine.

**Results:** In total, 97 inquiries addressing the use of antiemetics for NVP or HG during breastfeeding were identified. The following medications were addressed in the inquiries (*n* = 97): meclizine (51%), metoclopramide (33%), promethazine (16%), ondansetron (9%), and others (6%). The breastfed child was older than 6 months and 1 year in 96% and 71% of the inquiries, respectively. There was a preponderance of general inquiries (unclear motivation/double checking) (64%); however, one-third of the inquiries were generated by restrictive information from sources such as product information.

**Conclusion:** Based on our small review of spontaneous inquiries, there seems to be an information need about the use of antiemetics during lactation among women breastfeeding an older infant whilst suffering from NVP or HG. Addressing such use in guidelines for NVP and HG and/or other easily available information sources may be considered in order to balance out the restrictive information provided by the manufacturers. This could avoid potential unnecessary weaning of breastfeeding in an otherwise challenging situation.

## Introduction

Nausea and vomiting during pregnancy (NVP) is common and affects approximately 70% of the pregnant population (Fejzo et al., 2019). Hyperemesis gravidarum (HG) is the most severe form of NVP, and estimated rates among pregnant women vary from 0.3% to 10.8% (Fejzo et al., 2019). The majority of women with NVP can be managed with dietary and lifestyle changes, but more than one-third of patients experience clinically relevant symptoms that may require fluid and vitamin supplementation and/or antiemetic therapy (Fejzo et al., 2019). HG has been associated with poor maternal, fetal, and child outcomes, and is the leading cause of hospitalization in the first trimester and the second most common indication for pregnancy hospitalization overall (Fejzo et al., 2019). Having had a previous HG-pregnancy greatly increases the risk of experiencing HG in a subsequent pregnancy. Reported recurrence rates range from 15% to 81% (Fejzo et al., 2019).

SafeMotherMedicine is a medicines information service directed at pregnant and breastfeeding women in Norway (Heitmann and Schjøtt, 2020). We have noticed that we regularly receive inquiries about the use of antiemetics for NVP or HG from women who have become pregnant again while still breastfeeding. Although well-established treatment guidelines for NVP and HG exist (Royal College of Obstretricians and Gynecologists, 2016; Trovik et al., 2020), these guidelines do not address the use of antiemetics for NVP/HG in women who are still breastfeeding. Very little is also found in other literature (Breastfeeding and Medication, 2023; Woodstein, 2023), and no studies on the topic have been identified. Our objective was to describe the need for lactation risk information among women with NVP or HG, based on a review of spontaneous inquiries to our medicines information center. We also aimed to discuss points to consider when evaluating such inquiries and to raise awareness of this topic to promote further discussion among the medical community.

## Methods

In order to investigate lactating women’s information need on antiemetics, we conducted a review of the inquiries received by the web-based medicines information service for pregnant and breastfeeding women in Norway, SafeMotherMedicine (Heitmann and Schjøtt, 2020). In SafeMotherMedicine, all inquiries received through the web-based service and their corresponding answers are stored in a full-text database, searchable by staff only. The inquirer index the question with the relevant condition (e.g. pregnancy, breastfeeding, or both pregnancy and breastfeeding). The inquirer is also asked to describe medications, indication, dosage and duration of treatment, previous communication with the physician, the level of breastfeeding (exclusive or partial), and if the breastfeed child is healthy, sick, or premature. The medications in question are indexed by staff according to the Anatomical Therapeutic Chemical (ATC) classification system for medications (WHO Collaborating Centre for Drug Statistics Methodology, 2023) before the inquiry, and its corresponding answer, is stored in the database.

The SafeMotherMedicine database contains more than 35,000 spontaneous inquiries. The database was searched for inquiries concerning antiemetics for NVP or HG during breastfeeding received in the period June 2011 through 27 May 2022. This was conducted through a free text search on the Norwegian terms for NVP and HG, in combination with lactation as the indexed category. Search terms and indexed categories were combined using the Boolean operators (AND/OR) in the database. The retrieved inquiries were manually inspected to exclude inquiries not concerning NVP or HG (e.g. inquiries concerning the use of antiemetics for travel sickness) or duplicate cases. Inquiries not concerning medications were excluded. The authors analyzed the included inquiries through a combination of text inspection and evaluation of the associated indexation in the database. The inquiries were examined and categorized according to the following: 1) medicine name (ATC-code); 2) timing of medicine use with respect to breastfeeding: ongoing use/asking before use/during planning of breastfeeding/not revealed; 3) reason for asking SafeMotherMedicine: general inquiry (unclear motivation and/or double checking)/physician advised against use/product information advised against use/other sources advised against use/other or unclear reason; and 4) age of the breastfed child: <6 months/7–11 months/≥1 year/not revealed.

## Results

**FIGURE 1:**
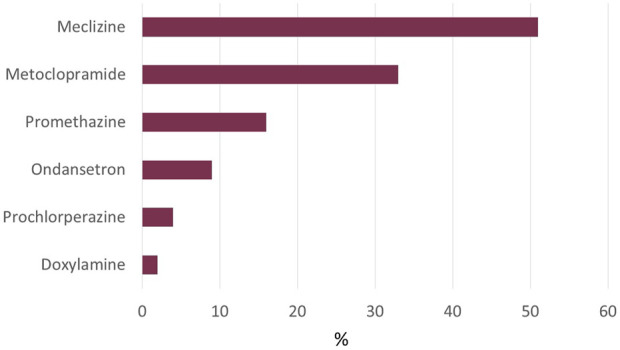
Antiemetics involved in inquiries about the use during breastfeeding in women with NVP/HG. Abbreviations: NVP, nausea and vomiting during pregnancy; HG, hyperemesis gravidarum. The total number exceeds 100% as some inquiries address more than one medication.

In the SafeMotherMedicine database, 150 inquiries were identified using the primary search. In total, 53 inquiries were excluded from further analyses due to other indications, not concerning medications or duplicate cases. This resulted in 97 included inquiries addressing the use of antiemetics for NVP or HG during breastfeeding. Most inquiries were regarding meclizine (51%), followed by metoclopramide (33%), promethazine (16%), ondansetron (9%), prochlorperazine (4%), and doxylamine (2%) (Figure 1). SafeMotherMedicine was most often consulted before medicine use (63%), and in 49% of the inquiries, the woman stated that she already had discussed the use with a physician.

**FIGURE 2:**
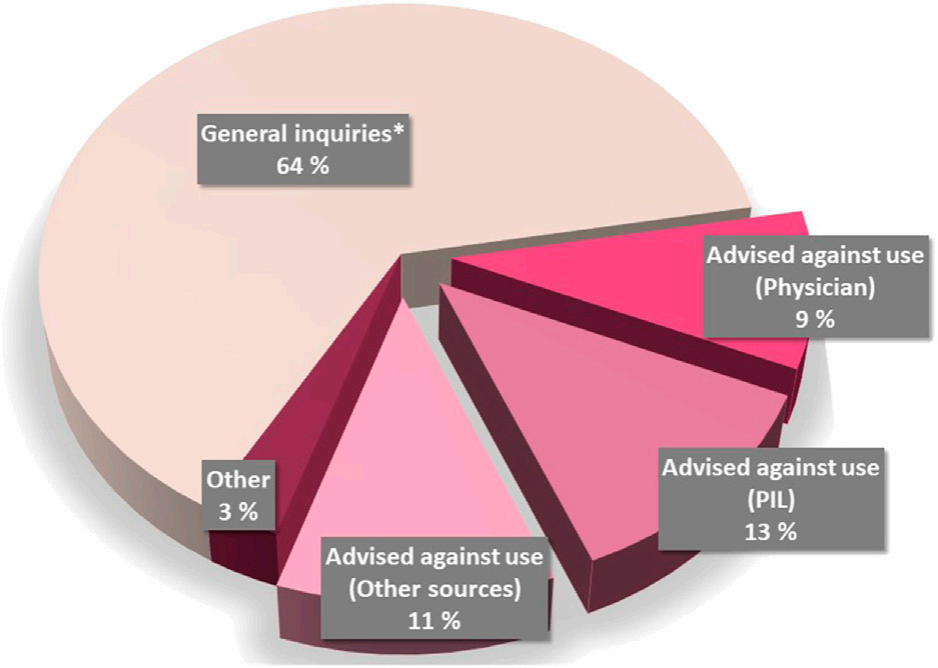
Motivation for asking SafeMotherMedicine about the use of antiemetics for NVP/HG during lactation. *General inquiries include those in which the women wanted to double-check the advice already received, or the motivation for asking about the use was not revealed. Abbreviations: PIL, patient information leaflet; NVP, nausea and vomiting during pregnancy; HG, hyperemesis gravidarum.

**FIGURE 3:**
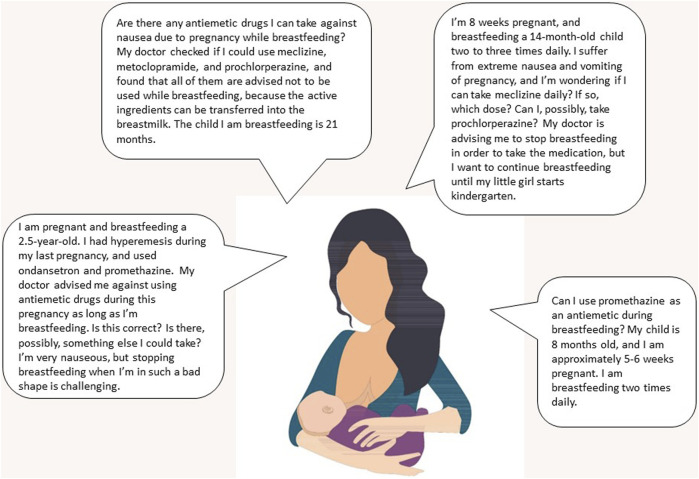
Examples of inquiries to SafeMotherMedicine, a national medicines information service for pregnant and breastfeeding women, about the use of antiemetics for NVP/HG from women who are breastfeeding. Abbreviations: PIL, patient information leaflet; NVP, nausea and vomiting during pregnancy; HG, hyperemesis gravidarum. Image credit: Colourbox.com.

Among the inquiries in which the infant age was revealed (*n* = 90, 93%), the breastfed child was older than 6 months and 1 year in 96% and 71% of the cases, respectively. There was a preponderance of general inquiries (64%), e.g. the women were double-checking whether the use during breastfeeding was safe or the motivation for asking was not revealed (Figure 2). However, one-third of the inquiries (*n* = 32, 33%) were generated by restrictive information, which was either provided by physicians (28%), or found in product information (38%) or other sources (34%) (examples are shown in Figure 3).

## Discussion

Our small study from a web-based service for pregnant and breastfeeding women in Norway suggests that there is a need for lactation risk information concerning antiemetics among women with NVP or HG. However, antiemetic use during breastfeeding is not addressed in Norwegian or international treatment guidelines, and we have not found any studies that explore this issue.

Breastfeeding is strongly promoted in Norway, and women are considerably encouraged to initiate and maintain breastfeeding, in line with the World Health Organization’s recommendations (World Health Organization, 2023). Furthermore, in more severe NVP and HG, there is a clear need for pharmacotherapy (Fejzo et al., 2019; Trovik et al., 2020). For women still breastfeeding when becoming pregnant and who develop HG, this can be challenging (Breastfeeding and Medication, 2023; Woodstein, 2023). It should be considered that to abruptly wean an older child in order to take antiemetics could be problematic (Breastfeeding and Medication, 2023).

In one-third of the inquiries, the contact with our service was caused by restrictive information, either from the manufacturer, healthcare professionals, or other sources. Our concern is that the restrictive information found in product information (The Norwegian Medicines Agency, 2022) could lead the woman to feel that she is left to choose between breastfeeding or the use of antiemetics. However, this contradiction is in our opinion unnecessary, especially in light of the fact that most of the infants will be older than 6 months of age. This is in accordance with one of the leading references on the use of medications during lactation, which has classified all the relevant antiemetics as either probably compatible or compatible with breastfeeding (Hale and Krutsch, 2023). As data on transfer to breastmilk are lacking for most of the antiemetics, other reference literature is more restrictive, especially with respect to the use of repeated doses and/or the use in premature infants or in the neonatal period (The Norwegian Medicines Agency, 2022; National Library of Medicine USA, 2023; E-lactancia, 2023). However, the short-term use is generally considered to be associated with little risk for the infant (The Norwegian Medicines Agency, 2022; Hale and Krutsch, 2023; National Library of Medicine USA, 2023; E-lactancia, 2023).

As metoclopramide has been used as a galactagogue to increase breastmilk production, data are available. A variable degree of transfer has been documented for metoclopramide; however, the relative infant dose (RID) is expected to be under 10% in most cases (National Library of Medicine USA, 2023). Its use during breastfeeding is considered compatible by independent sources (Hale and Krutsch, 2023; National Library of Medicine USA, 2023; E-lactancia, 2023). With respect to ondansetron, a computer model that simulated ondansetron levels in breastmilk estimated the amounts in milk to be much lower than the therapeutic dose recommended for infants (National Library of Medicine USA, 2023; E-lactancia, 2023; Job et al., 2022). For other antiemetics, small amounts are, in general, expected to transfer either based on pharmacokinetic properties or data from other related medications (Hale and Krutsch, 2023; National Library of Medicine USA, 2023; E-lactancia, 2023; Ngo et al., 2022). Doxylamine is expected to transfer to, breastmilk. According to one of the leading references on the use on medications during lactation, the use of the doxylamine-pyridoxine combination product (10 mg doxylamine/10 mg pyridoxine) in typical dosing for NVP would seem acceptable in lactation as long as the infant is monitored for antihistamine-related side effects (Hale and Krutsch, 2023).

According to pediatric references, there is experience with use in pediatrics (infants and/or toddlers) for most of the relevant antiemetics (The Norwegian Medicines Agency, 2022; KOBLE, 2023; Paediatric Formulary Committee, 2023). The exception is doxylamine; however, antihistamines are considered to have a relatively mild side-effect profile when used in recommended dosages.

The fact that the majority of the breastfed children in question were 1 year or older, and that nearly all children were older than 6 months, is also a mitigating factor as these children are far past the most vulnerable period with respect to the risk of experiencing adverse reactions of medications in breastmilk. Over half of all such reported adverse events occur within the first 4 weeks of age and 80% during the first 8 weeks (Hale and Krutsch, 2023). Older infants are at somewhat lower risk due to higher metabolic capacity (Hale and Krutsch, 2023). In addition, as the child becomes older, breastmilk constitutes a steadily decreasing share of the child’s nutritional intake, in favor of solid foods. It is estimated that after 12–18 months *postpartum*, the total milk ingested by the infant drops significantly (20–100 ml/day) (Hale and Krutsch, 2021). Furthermore, most women find that their milk supply diminishes as the pregnancy progresses, also limiting the amount of breastmilk an infant will receive from a pregnant mother (Royal College of Obstretricians and Gynecologists, 2016; López-Fernández et al., 2017).

As the child gets older, the detection of potential adverse events, especially more general symptoms such as sedation, is presumed to be easier as the child is expected to have a more consistent circadian rhythm. Furthermore, the parents will know their child better and, therefore, be more equipped to detect symptoms deviating from the child’s normal behavior or health status. In general, infants receiving breastmilk once or twice a day are considered to be at a very low risk (Hale and Krutsch, 2023).

In conclusion, based on our review of spontaneous inquiries, there seems to be an information need about the use of antiemetics during lactation among women breastfeeding an older infant whilst suffering from NVP or HG. However, further studies are needed both to estimate the prevalence of use of antiemetics during lactation and to identify the information need. Most antiemetics have limited documentation on use during breastfeeding, which may explain why one-third of the inquiries were generated by restrictive information. However, commonly used antiemetics do not appear to pose an unacceptable risk to breastfed children older than 6 months. The choice of an antiemetic should, therefore, in our opinion, rely on the women’s symptoms and perceived effect and not be restricted by breastfeeding and lack of data. Thus, we hereby propose an update of guidelines for NVP and HG and other easily available information sources to address the use of antiemetics during lactation.

## Data Availability

The original contributions presented in the study are included in the article; further inquiries can be directed to the corresponding author.
